# Do housing rental and sales markets incentivise energy-efficient retrofitting of western Germany’s post-war apartments? Challenges for property owners, tenants, and policymakers

**DOI:** 10.1007/s12053-023-10102-y

**Published:** 2023-03-28

**Authors:** Ray Galvin

**Affiliations:** 1 Cambridge Institute for Sustainability Leadership, University of Cambridge, 1 Regent Street, Cambridge, CB2 1GG UK; 2 Institute for Future Energy Consumer Needs and Behavior (FCN), School of Business and Economics/E.ON Energy Research Center, RWTH Aachen University, Mathieustrasse 10, 52074 Aachen, Germany

**Keywords:** Western Germany’s post-war apartments, Market for energy efficiency, Sales and rental premiums, Economic viability, Policy interventions

## Abstract

A post-World War 2 building boom in western Germany (the original *Bundesrepublik*) produced a cohort of some 8 million apartments, built in 1946–1979, that are relatively homogeneous in design and materials. On average, these apartments are very energy-inefficient, consuming around 147 kWh of heating energy per square meter of floor area per year (kWh/m^2^/y). Retrofitting them to about 50 kWh/m^2^/y is necessary to meet Germany’s climate goals. Considerable skill and infrastructure have developed to attempt to achieve this, but it is expensive. This study investigates whether sales and rental markets disincentivise property owners from retrofitting these apartments to high energy efficiency standards. Data from sales and rental advertisements in 2019–2021 in Germany’s largest online housing advertisement portal, Immoscout24, were used to estimate market sales and rental premiums for energy efficiency in these apartments. For property owners who retrofit apartments then sell them, sales premiums for energy efficiency generally fail to compensate for the retrofit costs, unless the renovation is subsidised. Meanwhile, for purchasers, the reduction in energy costs due to higher energy efficiency does not compensate for the higher purchase price. Likewise, for landlords/landladies who retrofit apartments then rent them out, the rental premiums due to higher energy efficiency are nowhere near sufficient to compensate for the retrofit costs. Tenants, however, can often offset the rental premium through energy savings. In all four cases, there is regional variation. Based on a detailed investigation of this market for energy efficiency, this study suggests specific policy interventions to compensate for these market anomalies.

## Introduction

This paper investigates whether housing rental and sales markets in western Germany support the economic viability of energy-efficient retrofitting of apartments built during the reconstruction boom after the Second World War. The paper differs from existing studies in two main ways. First, it simultaneously investigates both rental and sales markets for energy efficiency in a particular cohort of residential buildings. Second, the cohort it targets is relatively homogeneous in terms of design, materials, use, and retrofit technology.

Most of these buildings have flat, plain façades, simple roof construction, minimally thin walls of concrete mixed with rubble, concrete floors between storeys, an accessible attic, and a useable basement. Most are of four storeys (though some have five, three, or two). They represented approximately 45% of western Germany’s total stock of apartments in the most recent comprehensive survey (Loga et al., 2015). Furthermore, these apartments are different in design and materials from those built in eastern Germany in the same period and can be seen as a building cohort in its own right (Scholz & Veenis, 2012).

As these apartments were constructed before the building code mandated energy efficiency standards, they are notoriously energy-inefficient. As noted below, their average estimated energy intensity is 147 kWh per square meter of floor area per year (kWh/m^2^/y). According to the German standard DIN V 18,599, which is used to assess energy intensity, this means that an apartment with the cohort’s average floor area of 82 m^2^ would consume 12,054 kWh/y to keep all rooms at a temperature no lower than 19 C all year round (DIN (Deutsche Institut für Normung), 2022). This is about 3.5 times the amount these apartments would be allowed to consume to meet Germany’s climate goals by 2040 (Galvin, 2022).

Because of these buildings’ reputation as architecturally simple, ubiquitous, and energy-inefficient, a vast energy efficiency refurbishment industry and infrastructure has developed which specialises in retrofitting them, along with considerable research and development. The standard refurbishment approach has been to painstakingly fit blocks of insulation material to the outer walls, cover this with render, replace the windows with double- or triple-glazed models, insulate the roof either above or between and under the rafters, insulate the basement ceiling, and modernise the heating system (Loga et al., 2015). Due to shortages of skilled labour and the need to accelerate the rate of refurbishment, a new approach known as “serial renovation” (*serielle Sanierung*) has recently developed. Here, lasers measure and map the exact form of the façade, and a new, energy-efficient stick-on layer is made off-site, including windows, then trucked to the building and affixed in a matter of days. Currently this technology is being trialled in cities such as Mönchengladbach, Stuttgart, and Bochum (Wohnungswirtschaft, 2020). Although the German Energy Agency maintains that this “allows existing buildings to be brought up to the climate-neutral Net-Zero standard quickly, easily and affordably” (dena (Deutsche Energieagentur) 2022), high costs are still a major problem. Profitability is currently dependent on substantial EU and German government subsidies, though proponents argue that costs will fall as serial renovation processes develop.

For simplicity, this paper divides western Germany’s apartments into four cohorts according to time of build: pre-1946; 1946–1979; 1980–2009; and post-2010. The pre-1946 cohort are heterogeneous and of low energy efficiency (average energy intensity 150kWh/m^2^/y), but usually very difficult to upgrade for higher energy efficiency. The 1980–2009 cohort represents the earliest years of mandated energy efficiency standards for new builds. By today’s standards, these are poor performers (average 112 kWh/m^2^/y) and heterogeneous in design, and there is little or no experience of improving their energy efficiency.^1^ The post-2009 cohort generally have high energy efficiency (average 46.5 kWh/m^2^/y). The immediate post-War cohort, taken here as 1946–1979, therefore represents the low-hanging fruit, as they are the easiest to retrofit and are among the most energy-inefficient.

In Germany, there is no requirement to upgrade properties for energy efficiency, though whenever 10% or more of any particular feature, such as a wall or roof, is being substantially repaired, the whole of that feature must be upgraded to the new-build standard. Hence, there is no external, legal pressure on property owners to upgrade. This study therefore asks whether there is *market* pressure to upgrade: do the sales and rental markets incentivise energy efficiency upgrading of this cohort of apartment buildings, or do they discourage it? In particular, what does the market imply for a property owner who upgrades then sells, or who upgrades then offers the property for rent, or who lives in the upgraded property, and what does the market imply for a buyer or tenant? Who benefits and who is disadvantaged, by how much, and what policy shifts might be needed to better align the market with the need to refurbish this stock of buildings fast enough to meet Germany’s climate goals?

The “Literature review” section of this paper positions this study alongside existing research in the same general area. The “Method” section explains the method of estimating the market for energy efficiency. The “Results/findings” section presents the findings and discusses these as they arise, and the “Conclusion and policy implications” section offers conclusions and implications for policy.

## Literature review

There is a broad consensus in research of the past four decades that higher energy efficiency in housing generally brings higher sales and rental prices. Johnson and Kasserman (1983) were among the first to investigate this, with empirical work in the US housing market. They found a positive correlation between energy efficiency and selling prices and posited some basic reasons for this. First, house purchasers usually plan to keep their house for long periods compared to other goods and are therefore inclined to calculate long-haul costs and benefits, including energy costs. Second, if credible information about energy efficiency and savings is available, this can motivate a potential purchaser to incorporate it in their deliberations. Third, energy prices were prominent in the news when the study took place—as they have again become today.

As Taruttis and Weber (2022a, 2022b) note, later in the same decade Dinan and Miranowski (1989) found similar results for house sales and energy efficiency in the US state of Iowa, and over the next decades, similar findings emerged in studies in Singapore (Deng et al., 2012), Japan (Fuerst & Shimizu, 2016), and the Netherlands (Brounen & Kok, 2011), where different countries’ housing markets used a variety of types of labels and indicators of energy efficiency. A study on rental premiums for office space in the Netherlands also showed a positive correlation with energy efficiency (Kok & Jennen, 2012).

Studies investigating the effects of the European Union’s Energy Performance Certificate (EPC) on sales prices were offered by Lyons et al. (2013), though this was not peer-reviewed, and a later study was offered by Jensen et al. (2016). Studies were offered for Ireland (Stanley et al., 2016), Sweden (Cerin et al., 2014), England (Fuerst et al., 2015), the Netherlands (Chegut et al., 2016), Portugal (Ramos et al., 2015) (not peer-reviewed), Spain (Marmolejo Duarte and Chen, 2019), Romania (Taltavull et al., 2017), the Norwegian rental market (Khazal & Sønstebø, 2020), the French housing sales market (Civel, 2020) (not peer-reviewed), and the rental and sales markets in Berlin (Kholodilin et al., 2017) and Germany as a whole (Cajias & Piazolo, 2013).

All these studies found a significant positive effect of energy efficiency on prices, though several recent studies have found this to be negligible, such as Olaussen and Solstad (2019) for Oslo, Norway, and Fregonara et al. (2017) for Turin, Italy. A further interesting effect in the UK was identified in a Bank of England study by Guin and Korhonen (2020): mortgage payments for properties with higher energy efficiency are less likely to go into default than for properties with lower energy efficiency. This is evidence of a substantial market premium for energy efficiency. Households who can afford a significant market premium tend to have higher, steadier incomes and are less likely to default on their debts.

The present study relates closely to recent studies of the German market, which focus on rental and sales premiums respectively. Cajias et al. (2019) investigate the effect of energy efficiency on the residential *rental* market for *all* residences in 403 districts across Germany. They regress the rental price against different grades of energy efficiency certificate (A, B, etc.) together with control variables including dwelling and local area characteristics. They conclude that property owners “obtain a small but significant green premium when leasing residential dwellings” but that this is “less pronounced across the seven major cities, Berlin, Hamburg, Munich, Frankfurt, Stuttgart, Cologne and Düsseldorf, possibly due to the strong demand for housing and the low supply of housing …” (op. cit.: 186, and compare with März, 2018 and März et al., 2022 for the Ruhr area). They also find that dwellings with low energy efficiency tend to take longer to rent out, so that higher energy efficiency makes a rental portfolio financially more liquid. Their findings imply that reducing energy consumption by 75 kWh/m^2^/y can lead to a rental premium of around 14 €/month per dwelling (1000 €/year for a block of 6 apartments). This amounts to just 4200 €/apartment over a 25-year lifetime of the energy efficiency measures, a very poor return on an investment of tens of thousands of euros in the energy efficiency upgrade.

On a smaller geographical scale, Taruttis and Weber (2022a) investigate rental premiums of apartments in the German state of North Rhine-Westphalia. This gives the advantages of a large population in a region with homogeneous climate and consistent regulation but heterogeneous socio-demographics. They find a small rental premium for higher energy efficiency of the building fabric (good insulation and windows), which is larger if the type of heating system is known to be inefficient. This interesting finding shows a similar dynamic to the results of a study by Møller and Martinello (2022) in the Danish housing market. These authors found that higher energy prices reduce the market value of houses that do not have central heating (a relatively cheap and efficient method of heating) and that energy-efficient renovation of the building fabric mitigates this, though not sufficiently to cover the costs of the renovations.

Regrading *sales* markets, Taruttis and Weber (2022b) use a large database of house sales throughout Germany for 2014–2018 to estimate sales cost premiums for energy efficiency for *detached houses*, of all construction periods, estimating this for each 1 km^2^ micro-region. They also compare sales price premiums with initial investment costs and estimate future energy cost savings. They find an average premium of 6.9% of the selling price for an energy intensity reduction of 100 kWh/m^2^/y. Using their implied figures of an average selling price of 307,520 €/m and average floor area of 155 m^2^, this equates to a sales premium for energy efficiency of 212.19 €/ per reduced kWh/m^2^/y, or 21,219 € for a house upgraded by 100 kWh/m^2^/y. This is far lower than typical upgrade costs of around 40,000–60,000 €.

These results could be interpreted to imply that the market is reflecting the *purchaser’s* costs and benefits and that it would reflect the *vendor’s* costs and benefits only if government subsidies were to defray about half the costs of the upgrade. However, the authors also note that there is a wide geographical disparity in the sales premium for energy efficiency, and in general the sales premium is higher than average in rural areas and substantially lower in large cities with high housing demand. They suggest this indicates that where housing demand is high, property owners who do not upgrade can charge high prices, whereas in areas with low housing demand a property owner who does not upgrade will find it much harder to sell a house.

The above studies bring important findings for Germany as a whole: energy efficiency upgrading generally brings sales and rental premiums; the magnitudes of these vary geographically; for detached houses in large conurbations with high housing demand the premiums for energy efficiency tend to be lower; the premiums for energy efficiency do not fully compensate vendors; and the premiums compensate landlords/landladies even less.

**Fig. 1:**
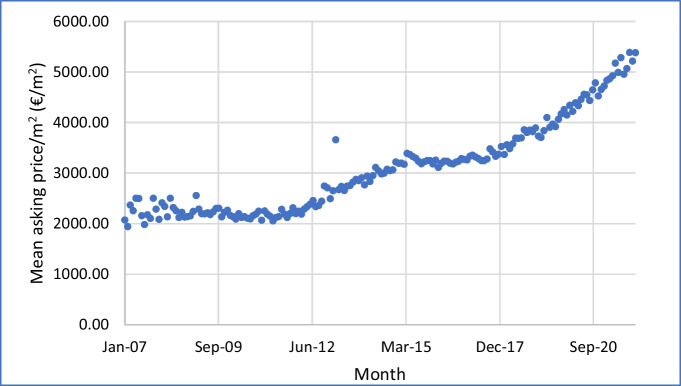
Mean asking price per square meter of floor area for apartments for sale, western Germany, Jan 2007–Dec 2021, by month of advert

**Fig. 2:**
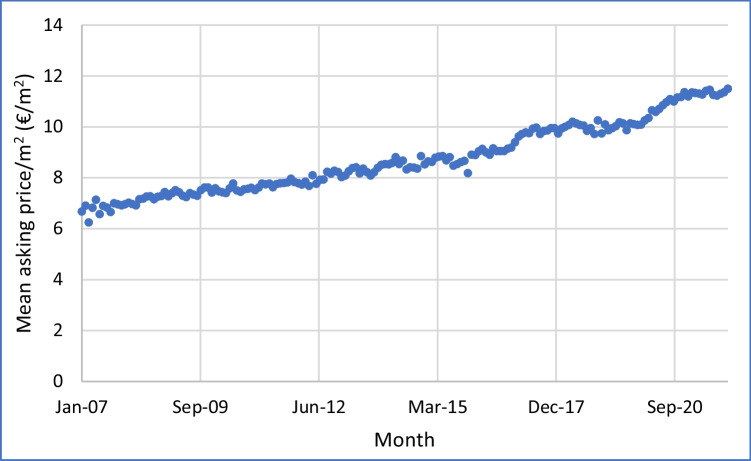
Mean asking price per month per square meter of floor area for apartments for rent, western Germany, Jan 2007–Dec 2021, by month of advert

**Fig. 3:**
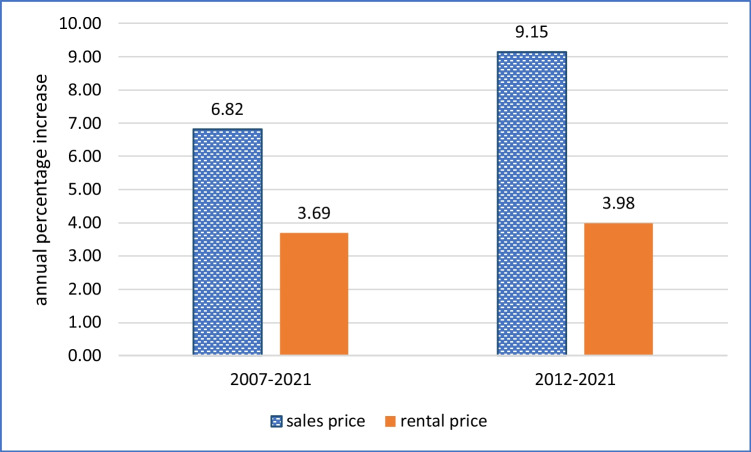
Comparison of annual percentage increases in sales and rental prices per square meter of floor area of apartments, western Germany, 2007–2021 and 2012–2021

A study by the German Central Bank (ter Steege & Vogel, 2021) investigates energy efficiency premiums from a broader perspective: the overall influence of increasing energy prices, due to climate policy, on Germany’s real estate market. They find that upward pressure on energy prices due to climate policy would put downward pressure on the market value of energy-inefficient properties, but would not increase the relative value of properties with higher energy efficiency. The word “relative” is important here, since property prices are increasing for other reasons (see Fig. 1, Fig. 2, and Fig. 3). The findings take on further relevance in 2022 since energy prices are now increasing markedly, for reasons other than climate policy.

A study by the Bank of England (Ferentinos et al., 2021) found a similar phenomenon for the UK housing market. Prices of carbon-intensive properties affected by climate policy decreased by about £5000 to £9000 relative to others.

The present study extends existing approaches in three ways. First, it focuses on one segment of the market, namely, apartments built in western Germany in the post-war reconstruction boom of the 1950s–1970s (more precisely 1946–1979). This is because, as noted in the “Introduction” section, these dwellings are energy-inefficient, relatively homogeneous in design, and relatively easy (but not cheap) to upgrade for energy efficiency, while there is a great accumulation of know-how and experience in upgrading.

Second, the study looks at both rental and sales premiums, the relationships between these two, and their relationships with energy efficiency retrofit costs and monetary savings through reduced energy consumption.

Third, the overall aim is to add to knowledge of the kinds of financial interventions in the market that would be required to stimulate large-scale, high-end energy efficiency renovations in this cohort of buildings.

The study therefore pursues the following research questions, with regard to 1946–1979-era apartments in western Germany:

1. How does high-end energy efficiency renovation influence the sales market value of these apartments?
2. How does it influence their rental market value?
3. To what extent does it pay back, through energy savings, and how is this related to market values?
4. How do these findings look from a purchaser’s standpoint?
5. How do these findings look from a prospective tenant’s standpoint?
6. How do they look from the perspective of a property owner who decides not to rent out but to live in the renovated property?

## Method

The study was based on detailed data from over a million advertisements of apartments for sale, and a further million-plus advertisements of apartments for rent, in Germany’s most-used real estate portal, Immoscout24,^2^ covering the years 2007–2021 but focussing particularly on advertisements in 2019–2021. Note that pre-2007 sales and rental data is not currently available from Immoscout24. The datasets had been extracted from Immoscout24’s records by RWI—Leibniz-Institut für Wirtschaftsforschung (commentary in Boelmann & Schaffner, 2020)—with datasets updated to December 2021. This comprises four datasets: apartments for sale, apartments for rent, houses for sale, and houses for rent. The first two of these only were used in this study, as its focus is on apartments only, both for sale and rent.

When clients upload an advertisement to Immoscout24, they fill in over 50 fields, covering topics such as energy rating, floor area, year built, number of bathrooms, and date of last renovation. There are two alternative types of energy rating a client can give: “Bedarf”, meaning the apartment’s theoretical heating energy intensity (kWh/m^2^/y), which is an objective assessment based on building substance, geometry, materials, etc.; and “Verbrauch”, which is the average heating energy consumed by the occupants over the past 3 years. Only advertisements giving the first of these were used in this study (unlike in Taruttis & Weber, 2022a, 2022b), as it is an objective indicator of the thermal quality of the apartment, irrespective of its current occupants’ heating behaviour, and can therefore have a direct influence on market values.

The databases were purged of repeat advertisements where the same apartment was advertised with the same characteristics more than once in any 6-month period. It was also purged of only the most extreme values, e.g. apartments advertised for sale for more than 20,000,000 €, due to the distortionary effect on regressions. Further purging took place dynamically during each regression or summation, using code to drop observations that had key variables missing. Note that the energy ratings are those used in the actual advertisements for sale and rent. They are therefore the energy ratings that the sales and rent markets are responding to. Whether or not the energy ratings are accurate therefore does not affect the market response directly. However, it is a very important question for its own sake (see reviews in Cozza et al., 2021) and is the subject of a further forthcoming paper. An assumption in the analysis is that potential purchasers and tenants respond to the energy ratings as given.

Ordinary least squares regression analyses were performed for advertisements inserted in 2019–2021, separately for sales and for rentals, to find the cost premiums and rental premiums which the markets were setting for improved energy efficiency. The years 2019–2021 were selected for the regression analyses because these are the most recent and therefore take into account the general market price increases of the previous 15 years.

The hypothesis was that each reduction in energy intensity of 1 kWh/m^2^/y would bring an increase in both the selling and rental prices which property owners could demand, in the market at the time of the advertisement. By comparing these figures with typical costs of energy efficiency renovations and the cost of heating energy in 2019–2021, conclusions could be drawn about how the sales and rental markets compared with each other and with these two factors.

In the regression analyses for apartment *sales*, the dependent variable was the asking price, and the independent variable of interest was the energy rating, given in in the database in kWh/m^2^/y. Note that this is the reciprocal of energy efficiency: the lower the energy rating, the higher the energy efficiency and therefore the lower the energy consumption. The analyses seek to find how much change in asking price is associated with a change of 1 kWh/m^2^/y in energy rating.

In the regression analyses for apartments for *rent*, the dependent variable is monthly basic rent, and again the independent variable of interest is the energy rating. Basic rent (*Kaltmiete*) is the portion of rent that the property owner gets, whereas supplementary rent (*monatliche Nebenkosten*) covers certain utilities such as rubbish collection and pavement snow sweeping and is collected by the property owner but passed on to these bodies. The aim of these analyses is to find how much change in *basic* rent is associated with a change of 1 kWh/m^2^/y in energy rating.

For both sales and rentals, other independent variables can influence selling or rental price, the most obvious being floor area. These can be seen as control variables. Including them enables us to find how much the energy rating influences the price (rental or sale) assuming all other influences are held constant.

For both sales and rents, a large number of preliminary regression analyses were performed using various combinations of independent variables, to find which combination gave the best fit to the data, i.e. the highest adjusted *R*-squared value for the regression model.

The distributions of the continuous variables “selling price”, “basic rent”, “supplementary rent”, “floor area”, and “energy rating” are right-skewed, since they are bounded by zero at the low end but theoretically open at the high end. Regressions using log transformations of these variables generally gave the best fit, and it is these results that are displayed below. However, linear regressions were also performed and the results recorded. Their regression results seldom differed from the linear transformations of the log–log regression results by more than 20%.

The linear models have the form:

1 $$P= {\beta }_{\mathrm{E}}.E+ {\sum }_{\mathrm{n}=1}^{N}{(\beta }_{\mathrm{n}}.n) +er+c$$

where *P* is the premium (rental or sales, depending on the dataset), *ß*_E_ is the coefficient of “energy rating”, *E* is the energy rating, *ß*_n_ are the regression coefficients of a matrix of *N* control variables, *er* is the error term, and *c* is a constant.

The log–log-linear models have the form:

2 $$\mathrm{log}\left(P\right)={\beta }_{E}^{^{\prime}} .\mathrm{log}\left(E\right)+{\beta }_{F}^{^{\prime}}.\mathrm{log}\left(F\right)+ {\beta }_{S}^{^{\prime}}.\mathrm{log}\left(S\right){\sum }_{\mathrm{m}=1}^{M}.{(\beta }_{m}^{^{\prime}}m) +er+c$$

where *ß’*_E_ is the coefficient of the log of the energy rating, *ß’*_F_ is the coefficient of the log of the floor area *F*, *ß’*_S_ is the coefficient of the log of the supplementary rent *S*, and *ß’*_m_ are the regression coefficients of a matrix of *M* other control variables.

To translate *ß’*_E_ into linear form, for example, for sales premium, we use:

3 $$P=-\frac{{S}_{a}}{{I}_{a}}\bullet {\beta }_{E}^{^{\prime}}$$

where *S*_a_ is the average sales price, *I*_a_ is the average energy rating, and *ß’*_E_ is the coefficient of log (*E*).

For each preliminary regression, a variance inflation factor (VIF) test was performed for multi-collinearity. Where there was high collinearity between two independent variables (such as floor area and number of rooms), i.e. VIF score > 3.0, the one with the lowest *t*-score was dropped. The surviving variables used in the regressions for *sale price* are given in the following list, with comments where appropriate:

- *Energy rating, in kWh/m*^*2*^*/y.* As noted above, a higher (worse) energy rating would be expected to correlate negatively with asking price. Although the cohort of interest is 1946–1979-era apartments, regression analyses were also performed for apartments from the other three cohorts: pre–1946; 1980–2009; and 2010–2021.
- *Months since January 2007.* Presumably, the more recent the advertisement, the higher the asking price (only the 36 months in 2019–2021 were used in the regressions, but the full range was used for some descriptive statistics).
- *Floor area, number of bathrooms, number of balconies.* These three variables would be expected to correlate positively with asking price.
- *Basement storage provided (a dummy variable) and parking available (a dummy variable).* Both these would be expected to correlate positively with basic rent.
- *Built-in kitchen (a dummy variable).* In Germany when apartments are bought or rented out, the previous occupants often dismantle the kitchen and take it with them to their new home—a practice which Anglo-Americans tend to find astonishing, and which causes enormous frustration and anger among German people due to wrangling over extra charges for leaving a kitchen intact, or in some cases for not leaving it intact (Kueche.de, 2022).
- *Big city.* a dummy variable for cities of population greater than 400,000, of which there are 12 in western Germany.
- *Medium city.* a dummy variable for cities of population between 200,000 and 400,000, of which there are 18 in western Germany.^3^

For the regressions against *rental price*, two additional independent variables were found to influence the fit of the model.

- *Supplementary rent.* As noted above, this is the extra rent which the property owner passes on the utilities concerned. It does not include energy costs. It is not clear why it correlates with the rent, but it could be that it acts as a proxy for the quality of the apartment or its neighbourhood.
- *Heating costs included in rent.* This is separate from “basic rent”, though it tends to correlate positively with the rental price. It means that the landlord/landlady collects the heating costs and passes them on to the energy provider

Unlike in Taruttis and Weber (2022a, 2022b), the variable *year of last upgrade* was not used in the regressions, because this gives no information about the extent of such an upgrade and can therefore be misleading. Also, the transaction costs are not given in the database, but these usually amount to about 12.5% of sale price in Germany and potential purchasers can calculate these based on sales price.

For both sales and rents, regressions were performed for apartments in all of western Germany together, as well as for each of the separate age cohorts of apartments noted above, i.e. pre-1946; 1946–1979; 1980–2009; 2020–2021. Further regressions were performed for “big cities”, “medium cities”, “rural”, and for each western German state that is not a city-state.

Further statistical analyses were performed to investigate related relevant issues such as sales and rental price increases over time and change of average energy efficiency rating over time.

Finally, it is sensible to ask whether sales and rental coefficients were influenced by the COVID-19 pandemic. The period covered by the regressions includes one pre-pandemic year and the two first years of the pandemic. To check whether the results are adversely affected by this, a separate set of regressions was performed for 2018–2019, the last two pre-pandemic years, and for 2020–2021, the first two years of the pandemic.

## Results/findings

### Descriptive statistics

Table 1 gives descriptive statistics for the 1946–1979-era apartments for *sale* in western Germany in 2019–2021, for the variables used in the regressions. Table 2 gives descriptive statistics for apartments for rent. Note the slight difference between the energy ratings, 147.5 kWh/m^2^/y compared to 146.5 kWh/m^2^/y, and floor area, 88.1 m^2^ compared to 78.4 m^2^. Since the data is population data (comprising all advertisements in the given time span, not a random sample of these advertisements), it can be interpreted as true differences, not statistical estimates. They indicate that there are slight differences between the populations of apartments for rent and apartments for sale, but these are very small.

**Table 1:** Descriptive statistics, apartments for sale in 2019–2021, 1946–1979 cohort, western Germany

Variable	Observations	Mean	Std. dev	Min	Max
Selling price (€)	15,259	293,195	236,474	10,000	4,795,000
Floor area (m^2^)	15,259	88.1	37.5	14	375
Bathrooms (no.)	15,259	1.1533	0.4180	0	5
Energy rating (kWh/m^2^/y)	15,259	147.5	56.6	8.6	397.8
Balconies (no.)	15,259	0.7874	0.4092	0	1
Month of advertisement	15,259	161.7	10.6	145	180
Built-in kitchen (dummy)	15,259	0.5117	0.4999	0	1
Basement storage (dummy)	15,259	0.7536	0.4309	0	1
Parking available (dummy)	15,259	0.9860	0.1173	0	1
Big city (dummy)	15,259	0.1943	0.3957	0	1
Medium city (dummy)	15,259	0.0879	0.2831	0	1

**Table 2:** Descriptive statistics, apartments for rent in 2019–2021, 1946–1979 cohort, western Germany

Variable	Observations	Mean	Std. dev	Min	Max
Basic rent (€)	28,748	707.7	347.7	70	3995
Supplementary rent (€)	28,748	159.6	69.7	0	495
Floor area (m^2^)	28,748	78.4	29.1	12	392
Bathrooms (no.)	28,748	1.0660	0.2606	0	5
Energy rating (kWh/m^2^/y)	28,748	146.5	59.7	5.8	399.3
Balconies (no.)	28,748	0.7489	0.4336	0	1
Month of advertisement	28,748	162.0	10.1	145	180
Built-in kitchen (dummy)	28,748	0.4420	0.4966	0	1
Heating costs in rent	28,748	0.6157	0.4864	0	1
Basement storage (dummy)	28,748	0.7568	0.4290	0	1
Parking available (dummy)	28,748	0.9636	0.1872	0	1
Big city (dummy)	28,748	0.1897	0.3921	0	1
Medium city (dummy)	28,748	0.1134	0.3171	0	1

To give a picture of how the market has developed over time, including the years prior to 2019–2021, Fig. 1 tracks the mean asking price, in euro per square meter of floor area, for these apartments in western Germany in each month of the period January 2007 to December 2021. We see stagnating prices from January 2007 until December 2011, the period of the Global Financial Crisis, followed by a steady upswing. The asking price increased from 2195 €/m^2^ in January 2012 to 5378 €/m^2^ in December 2021, an increase of 140%, or 9.15% per year cumulative. Over the whole period from January 2007 to December 2021, the increase was 169% or an annual cumulative increase 6.82% per year.

How does this compare with rental prices for apartments for rent? Fig. 2 indicates that rental prices increased steadily during the whole of 2007–2021, from 6.68 €/m^2^/month to 11.50 €/m^2^/month. This was 72.2%, or 3.69% per year cumulative. This is much lower than the annual average cumulative increase in sales prices over the same period, of 6.82% per year. It is even lower than the more recent annual increases in sales prices of 9.15%. A summary of these results is given in Fig. 3.

**Fig. 4:**
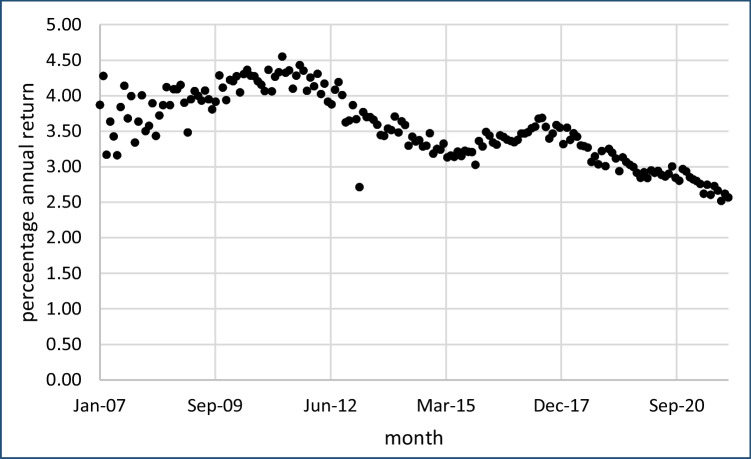
Annual percentage return on investment in rental apartments, western Germany, 2007–2021, by month of purchase and rental

This clearly indicates that renting out apartments of this cohort has become less and less profitable compared to their market sales value. Figure 4 displays this as the annual percentage return on investments in rental apartments (assuming that apartments rented and sold are of the same quality). The annual return reduced from 4.5% at the end of the Global Financial Crisis to 2.5% by December 2021.

The figure of an annual increase in rent of 3.69% is especially interesting since in Germany the basic rent can be increased by 4.77% per year (i.e. by 15% once every 3 years), and further large increases are permitted for energy efficiency improvements. Assuming the rental price of apartments coming onto the market is consistent with rents already being paid in apartments generally, it appears that the market is not allowing property owners to increase rents as much as is legally permitted.

These descriptive statistics raise an important question for property owners who retrofit to increase energy efficiency: can they make up the shortfall in rental profitability by increasing the energy efficiency of their properties? The regression results, below, help to answer this question.

### Regression results: premiums for energy efficiency

#### The sales premium for energy efficiency

Table 3 gives the results of regressions of key independent variables against asking price, for apartments listed *for sale* in western Germany in 2019–2021. The variable of interest is (the logarithm of) heating energy intensity, given in the table in bold type. Since both this and asking price are in log form, the regression coefficient can be interpreted as the percentage change in asking price for a 1% increase (sic) in energy intensity—hence, the coefficients are negative, indicating that a *reduction* in energy intensity is associated with an *increase* in price. The bottom row of the table translates this into the change in price, in euros, due to a reduction in energy intensity of 1 kWh/m^2^/y, using Eq. (3), thereby giving the sales premium for energy efficiency. For example, for the 1946–1979 cohort, each reduction of kWh/m^2^/y is likely to lead to an extra 361.60 € in asking price, on average.

**Table 3:** Apartments for sale in western Germany in 2019–2021, regressions of key variables against logarithm of asking price

Cohort (year built)		1946–1979 cohort	1980–2009 cohort	2010–2021 cohort	Pre-1946 cohort	Big city, 1946–1979 cohort	Medium city, 1946–1979 cohort	Small city and rural, 1946–1979 cohort
Log (floor area)	Coefficient	0.8796	0.9294	0.8938	0.8506	0.8384	1.0376	0.8668
	*t-statistic*	*80.17*	*89.8*	*166.39*	*31.908*	*34.3*	*26.54*	*66.95*
number of bathrooms	Coefficient	0.0582	0.0887	0.152	0.1588	0.1626	** − **0.0511	0.0360
	*t-statistic*	*5.57*	*9.12*	*37.62*	*7.203*	*6.85*	*-1.48*	*2.91*
**Log (energy intensity)**	**Coefficient**	** − 0.1819**	** − 0.1897**	**0.0397**	** − 0.3109**	** − 0.1820**	** − 0.2048**	** − 0.1790**
	***t-statistic***	*** − 20.13***	*** − 19.78***	***13.88***	*** − 18.026***	*** − 7.46***	*** − 6.83***	*** − 17.55***
Number of balconies	Coefficient	0.0580	0.0489	0.008	0.1893	0.1422	0.0769	0.0330
	*t-statistic*	*6.07*	*4.79*	*1.49*	*9.277*	*5.78*	*2.37*	*3.04*
Months since start	Coefficient	0.0104	0.0090	0.0092	0.0124	0.0083	0.0076	0.0111
	*t-statistic*	*28.79*	*25.84*	*57*	*14.126*	*9.53*	*6.41*	*26.68*
Built-in kitchen	Coefficient	0.1165	0.1489	0.046	0.0447	0.1687	0.1931	0.0931
	*t-statistic*	*15.21*	*19.51*	*11.65*	*2.337*	*9.04*	*7.54*	*10.54*
Basement storage	Coefficient	0.0246	0.0591	0.1383	0.0330	** − **0.1100	** − **0.0737	0.0647
	*t-statistic*	*2.72*	*6.93*	*37.77*	*1.614*	** − ***4.66*	** − ***2.34*	*6.36*
Parking available	Coefficient	0.0594	0.1789	0.036	** − **0.2002	** − **0.0289	0.1630	0.0690
	*t-statistic*	*1.83*	*3.14*	*0.91*	** − ***3.441*	** − ***0.52*	*2.19*	*1.43*
Big city	Coefficient	0.5187	0.6576	0.5694	0.7107			
	*t-statistic*	*52.84*	*64.19*	*145.08*	*29.180*			
Medium city	Coefficient	0.0057	0.1051	0.074	0.1479			
	*t-statistic*	*0.42*	*7.61*	*11.5*	*4.380*			
Constant	Coefficient	7.3762	7.2496	6.8457	7.8611	8.3698	7.3009	7.3169
	*t-statistic*	*81.83*	*71.7*	*128.27*	*36.820*	*38.55*	*26.76*	*67.17*
No. of observations		15,259	12,373	43,824	3,061	2,965	1,341	10,593
Adjusted *R*-squared		0.4756	0.6277	0.6775	0.5827	0.4685	0.492	0.3995
*F*		0.000	0.000	0.000	0.000	0.000	0.000	0.000
Hence, sales premium for each reduced kWh/m2/y		361.60 €	569.45 €	− 511.15 €	761.51 €	593.08 €	361.38 €	302.94 €

The first four regressions given in Table 3 cover the four different cohorts of apartments. The results show that of all the apartments built before 2010, i.e. before energy efficiency standards for new builds became very stringent, the 1946–1979 cohort has the lowest sales price premium for energy efficiency, at 361.60 € per kWh/m^2^/y of reduced energy intensity. This is probably because these are the cheapest and easiest to retrofit. The oldest cohort, built pre-1946, brings a much higher premium for energy efficiency, at 761.51 €/(kWh/m^2^/y). This is probably because these are far harder to retrofit, mostly due to their elegant three-dimensional façades, complex roof designs and high ceilings. The energy efficiency premium for apartments in the 1980–2010 cohort is 569.45 €/(kWh/m^2^/y), about 60% higher than for the 1946–1979 cohort. This is probably also because these are more heterogeneous and difficult to retrofit, and are already relatively energy efficient, at 112 kWh/m^2^/y, beyond which each kWh/m^2^/y of further reduction in energy intensity becomes extremely expensive.

The sales premium for the 2010–2021 cohort is anomalous, at − 511.15 €/(kWh/m^2^/y), suggesting that these properties bring a negative return for each reduced kWh/m^2^/y. Further regressions using these databases, and also using databases of semi-detached and detached houses for sale (in a forthcoming study), show a similar anomaly for the most energy-efficient dwellings. A finer grained examination shows that the effect for the 2010–2021 cohort is dominated by the very large number of new apartments coming onto the market in very recent years, most of which have energy ratings of around 20–30 kWh/m^2^/y. It seems that the shift from the so-called “EH-55” standard of around 27.5 kWh/m^2^/y to the even higher “EH-40” standard of around 20 kWh/m^2^/y actually brings a negative market return.

In any case, these apartments are not directly relevant to this study, which investigates the effects of energy efficiency retrofitting on sales and rental markets.

Note that *p* values are not shown in Table 3 (nor in Table 4) because the data is of whole populations, not random samples. *P* values are therefore irrelevant and misleading (in fact, almost all are 0.000 due to the large number of observations^4^). Instead, t-statistics are shown because their absolute value gives an approximate indication of the relative influence of each of the independent variables, on the dependent variable. For the 1946–1979 cohort, for example, the variable “log (floor area)” has the largest t-statistic by far, indicating that floor area is the biggest influence on asking price.

**Table 4:** Regression results for apartments for rent in western Germany in 2019–2021, regression of key variables against log (basic rent)

Cohort (year built) + A2:H26		1946–1979 cohort	1980–2009 cohort	2010–2021 cohort	Pre-1946 cohort	Big city, 1946–1979 cohort	Medium city, 1946–1979 cohort	Small city and rural, 1946–1979 cohort
Log (supplementary rent)	Coefficient	0.1041	0.1866	0.24897	0.2022	0.1691	0.0957	0.0847
	*t-statistic*	*18.99*	*31.98*	*100.34*	*21.46*	*11.07*	*5.89*	*13.85*
Log (floor area)	Coefficient	0.7223	0.6681	0.62119	0.6989	0.6435	0.8378	0.7326
	*t-statistic*	*127.12*	*114.54*	*225.98*	*67.09*	*43.57*	*48.74*	*113.08*
**Log (energy intensity)**	**Coefficient**	** − 0.0907**	** − 0.0716**	** − 0.04007**	** − 0.1298**	** − 0.0775**	** − 0.1142**	** − **0.0900
	***t-statistic***	*** − 26.53***	*** − 18.19***	*** − 33.47***	*** − 24.59***	*** − 7.97***	*** − 11.56***	*** − 23.65***
No. of bathrooms	Coefficient	0.0911	0.1006	0.09237	0.0912	0.1169	0.1172	0.0696
	*t-statistic*	*14.35*	*18.38*	*46.06*	*9.56*	*7.53*	*7.07*	*9.26*
Number of balconies	Coefficient	0.05876	0.03299	0.03002	0.1286	0.05360	0.06134	0.05543
	*t-statistic*	*15.56*	*7.08*	*12.47*	*19.88*	*5.23*	*6.51*	*12.74*
Months since start	Coefficient	0.00313	0.00259	0.00217	0.00495	0.00290	0.00296	0.00326
	*t-statistic*	*20.3*	*16.22*	*31.16*	*17.64*	*6.96*	*7.31*	*18.5*
Built-in kitchen	Coefficient	0.1545	0.1679	0.08590	0.1210	0.2332	0.2056	0.1260
	*t-statistic*	*48.19*	*49.47*	*59.38*	*20.46*	*26.87*	*23.7*	*34.54*
Heating costs in rent	Coefficient	0.08437	0.03342	** − **0.00678	** − **0.00849	0.09176	0.11639	0.07596
	*t-statistic*	*20.2*	*7.33*	** − ***3.15*	** − ***1.05*	*8.19*	*10.54*	*15.83*
Basement storage	Coefficient	0.00114	0.02118	0.02647	0.0090	** − **0.01852	** − **0.04402	0.00983
	*t-statistic*	*0.31*	*5.36*	*16.05*	*1.44*	** − ***1.83*	** − ***4.04*	*2.37*
Parking available	Coefficient	** − **0.02999	** − **0.01121	0.02772	** − **0.0883	0.05105	** − **0.05717	** − **0.06032
	*t-statistic*	** − ***3.56*	** − ***0.64*	*2.9*	** − ***6.34*	*2.81*	** − ***3.07*	*-5.53*
Big city	Coefficient	0.2905	0.3194	0.32527	0.3992			
	*t-statistic*	*69.18*	*71.6*	*185.09*	*53.56*			
Medium city	Coefficient	0.01956	0.06306	0.06418	0.1004			
	*t-statistic*	*3.88*	*10.59*	*25.78*	*10.52*			
Constant	Coefficient	2.4886	2.3236	2.34082	2.1074	2.6271	2.1984	2.5824
	*t-statistic*	*67.51*	*55.23*	*129.66*	*30.94*	*27.18*	*21.48*	*61.05*
No. of observations		28,704	24,623	90,439	8,873	5,442	3,252	20,010
Adjusted *R*-squared		0.647	0.6894	0.7231	0.7459	0.6188	0.734	0.6224
F		0.000	0.000	0.000	0.000	0.000	0.000	0.000
**Hence, rental premium for each reduced kWh/m2/y**		**0.4400 €**	**0.0716 €**	**0.8493 €**	**0.7668 €**	**0.4959 €**	**0.5068 €**	**0.4046 €**

From the vendor’s point of view, the coefficients indicate what features bring higher selling prices. A higher price is achieved for an apartment with a larger floor area, a lower (better) energy intensity rating (apart from the 2010–2021 cohort), a balcony, an extra bathroom, a built-in kitchen, basement storage, and parking availability. The positive coefficient of “months since start” indicates that the market became more lucrative by the month in 2019–2021.

The interpretation of the non-log variables’ regression coefficients is that a change of 1 unit in the variable is associated with a change in the logarithm of selling price equal to the value of the coefficient. For example, using Eq. (4), selling an average priced apartment in the 1946–1979 cohort 1 month later would increase the price from 293,195€ to 296,260€.^5^

4 $${P}_{n}= {e}^{(R+ \mathrm{log}{P}_{a})}$$

where *P*_n_ is the new selling price*, R* is the regression coefficient, and *P*_a_ is the average selling price.

The last three columns of Table 3 give results of separate regressions for the 1946–1979 cohort of apartments, in big cities (population > 400,000), medium cities (population 200,000–400,000), and less populous regions (cities < 200,000 and rural). Big cities show a relatively high sales premium for energy efficiency, at 538.08€ per reduced €/kWh/m^2^/y. Medium-sized cities show a more modest premium, at 361.38€, followed by smaller cities and rural areas, at 302.94 €. This might appear counter-intuitive, since high housing demand in the larger cities could make any apartment sell well, regardless of its energy efficiency. On the other hand, real estate in larger cities in Germany is in such demand that property owners can charge a premium for the improvements they make (and see further discussion below).

#### The rental premium for energy efficiency

Table 4 gives the results for a set of regressions of key independent variables against rental price, for apartments listed for rent in western Germany in 2019–2021. The same comments as above apply here to *t*-statistics and the absence of *p* values (again, most of which are 0.000).

Again, the sign and magnitude of the coefficients are in line with what would intuitively be expected, except for those with relatively small t-statistics, which are not very strong determinants of rental prices. The main interest here lies with the correlation between heating energy intensity and rental price. For all building cohorts, the sign of the regression coefficient of log (energy intensity) is negative: the higher (worse) the energy intensity, the lower the rent. For the cohort of most interest, the relatively easily upgradable apartments built in 1946–1979, the coefficient translates to a rental premium of 0.4400 € per reduced kWh/m^2^/y. The rental premium for pre-1946s apartments is higher, at 0.7668€ per reduced kWh/m^2^/y, probably for the same reasons as noted above regarding sales premiums: the pre-1946 apartments are much harder to retrofit. The premium also suggests there is high demand for older, elegant apartments with high energy efficiency.

The rental premium for energy efficiency in the most recently built apartments (2010–2021) is also high, at 0.8493 € per reduced kWh/m^2^/y, while the premium for the 1980–2009 cohort is very low, at 0.0716 € per reduced kWh/m^2^/y.

Again, the last three columns of Fig. 4 show regressions for 1946–1979-era apartments in big cities, medium-sized cities, and other regions, respectively. There is hardly any difference between the premiums for big and medium sized cities, with both close to 0.5000 € per reduced kWh/m^2^/y. For small cities and rural regions, the premium is some 20% lower, at 0.4046 €. This differs from the finding of Cajias et al. (2019) for rental dwellings of *all* types of buildings throughout the *whole* of Germany, confirming the value of investigating this particular, relatively homogeneous cohort of post-war western German apartments in its own right.

### Will this market support energy-efficient renovation among 1946–1979-era apartments?

In light of the above results, we now consider a property owner who has recently renovated a 1946–1979-era apartment to a high standard of energy efficiency, and a prospective buyer and tenant for such a property. Will the property owner get their money back if they sell it; rent it out; or keep it and live in it? Will a prospective tenant or the prospective purchaser get their money back, through energy savings, if they rent it or buy it? The average energy rating of this cohort of apartments is 147 kWh/m^2^/y. To reach the standard required to robustly support Germany’s climate goals would require a renovation to under 50 kWh/m^2^/y, meaning a reduction in heating consumption of about 100 kWh/m^2^/y for the average 1946–1979-era apartment.

#### Selling the apartment

Typical costs of energy efficiency renovation for this cohort of apartments to a high standard are between 400 €/m^2^ and 600 €/m^2^ of floor area, equating to between 32,000 € and 48,000 € for an apartment of 80 m^2^ floor area (Energiesparen, 2022). An upgrade to the exceptionally high standards now being considered by policymakers would be even more expensive (Galvin, 2022).

As seen in Table 3 column 1, the average sales premium is 361.60 € for each reduction of 1 kWh/m^2^/y (the figure from the linear–linear regression was 395.90 € for each reduction of 1 kWh/m^2^/y), suggesting a premium of 36,160 € for an improvement of 100 kWh/m^2^/y. On average, then, a vendor could get around 90% of their money back if they sell after retrofitting by this amount. Federal subsidies from the German Development Bank (*Kreditanstalt für Wiederaufbau*—KfW) for energy efficiency renovation would almost certainly cover the shortfall. This would also be the case for each of the types of regions: big cities, medium-sized cities, and small cities/rural areas. This is of course only an average, and the situations for different Federal states are considered below.

#### Renting the apartment out

The market is less supportive for renting the apartment out. The tenant’s premium of 0.440 €/(kWh/m^2^/y) (compared to 0.4619 €/(kWh/m^2^/y in the linear regression) means they will pay 44.00 €/month extra for an apartment that is 100 kWh/m^2^/y better than the norm. This amounts to a total of 13,200€ over the 25-year lifetime of the retrofit measures. This falls far short of the 40,000€ the vendor would have paid for the retrofit, or even 20,000€ if the vendor got federal subsidies amounting to 50% of costs. Looking at it another way, it would take almost 76 years for the retrofit to pay back, without subsidies.

There is of course the caveat that the rent will increase over time, and with it the rental premium. If the property owner demands the legal maximum basic rent increase of 15% every 3 years, the total rental premium received after just 34 years will amount to 41,026 €, sufficient for full payback. However, referring back to the discussion on Fig. 2, the actual average annual rent increase was only 3.69% in the years 2007–2021. At this rate, the rental premium will take 38 years to amount to 40,000€. A property owner thinking of retrofitting such an apartment to a high energy efficiency standard then renting it out might reason that the money could be better invested elsewhere. For example, as noted above in relation to Fig. 2, simply investing in additional property would bring a return of somewhere between 6.82% and 9.15% per year through capital gain.

It is fair to say, then, that a landlord/landlady who has retrofitted for energy efficiency is highly unlikely to get their money back through the market rental premium but might do so if they increase the rent persistently and have accessed generous federal subsidies. In other words, the market, in itself, does not support retrofitting before renting out, and subsidies might not be sufficient to make up for this lack of support.

Furthermore, according to Germany’s Civil Law 559 (*Bürgerliches Gesetzbuch §599*), property owners who do energy efficiency renovation on an already-rented property are allowed to increase the annual rent by 8% of the costs of the renovation, provided this does not increase the monthly basic rent by more than 3 €/m^2^/month, or 2 €/m^2^/month if the basic rent is less than 7 €/m^2^/month. For a retrofit costing 40,000 €, the legal limit of 8% is 3,200 €/y, or 267 €/month, which for an apartment of 80 m^2^ floor area equates to 3.34 €/month, so the average legally permitted increase would be 3.00 €/m^2^/month, or 240 €/month.

However, as noted above, the market only supports a rent increase of 44.00 €/month, falling far short of what the property owner can *legally* charge.

#### Keeping the apartment and living in it

What if the property owner keeps the apartment and tries to recoup their investment through energy savings gained by the higher energy efficiency? During the period covered by the advertisements, 2007–2021, the price of household natural gas stayed close to 6 eurocents/kWh (Verivox, 2022). Although it has recently gone much higher, the market in the period analysed, 2019–2021, was reflecting the costs in that period, so the rental and selling premiums for energy efficiency need to be seen in that light. A saving of 6 eurocents/kWh due to an energy efficiency upgrade of 100 kWh/m^2^/y for an apartment of 80m^2^ floor area amounts to an annual saving of 40 €/month, or 480 €/y, giving a total saving of 12,000 € over the 25-year lifetime of the retrofit measures. This falls far short of the 40,000 € it would have cost to do the retrofit. The gas price would need to be at least 20 eurocents/kWh to make the retrofit pay for itself, without subsidies.

This should not be surprising, since there is a long stream of literature showing that energy-efficient renovation in Germany does not pay for itself through energy cost savings, except possibly at the most modest levels of retrofitting (Galvin, 2010, 2014; März, 2018). It is also well known that modest retrofits, which bring reductions of only around 50 kWh/m^2^/y, are far more economically efficient than stringent retrofits, and that the cost of each kWh/m^2^/y reduction beyond this level increases exponentially (Conci et al., 2019).

This will change, however, with large increases in energy prices. At the time of the advertisements, a property owner, purchaser, or renter would have had no reason to think gas prices were likely to rise much above 6 eurocents/kWh, as they had hovered around this level for 16 years (Verivox, 2022). Since the war in Ukraine, however, gas prices have risen sharply, with some new customers having to pay up to 16 cents/kWh, and the German government now moving to cap them at 12 cents/kWh. Prices will not necessarily stabilise at the level of 20 eurocents/kWh that would be needed to make a stringent retrofit pay for itself without subsidies. There are good reasons to believe that the present price peak will last at least until the end of 2023, but there is strong political incentive to bring prices down (European Commission, 2022).

At the same time, there are sharp increases in the costs of energy-efficient renovation in Germany (McMakler, 2022), and these will offset much of the saving achieved by reduced energy consumption, for future retrofits. A property owner can therefore not be confident of recouping the cost of stringent energy efficiency renovation by saving on energy use. They can, however, “future-proof” their home against future sharp increases in energy and retrofitting costs, by retrofitting sooner rather than later.

#### The tenant’s perspective

How does this look from the tenant’s point of view? As noted above, on average a new tenant who rents an apartment in the 1946–1979 cohort pays a rental premium of 0.44 €/month for each reduced kWh/m^2^/y of energy intensity. For a reduction of 100 kWh/m^2^/y, this amounts to 44.00 €/month. This is more, but not much more, than the 40 €/month which the tenant will have saved in energy bills (at the 2019–2021 rate of 0.06 /kWh). Given that the rental premium figure of 44.00 €/month is merely an average and that there is substantial variation in the rental premium from region to region (see also below) and from building to building, it appears that some subsections of the market support the tenant’s interests well, but not others. On average the market falls short of supporting the tenant’s interests, but not far short.

For apartments in the pre-1946 and post-1979 cohorts, the tenant’s position is even weaker, as the rental premiums are substantially higher.

Furthermore, although future increases in the gas price will close this gap for apartments that are already rented out, they are unlikely to do so for apartments coming onto the market in the years after 2019–2021. The market is likely to adapt by increasing the rental premium for energy efficiency, as tenants, on average, become willing to pay more for it.

**Fig. 5:**
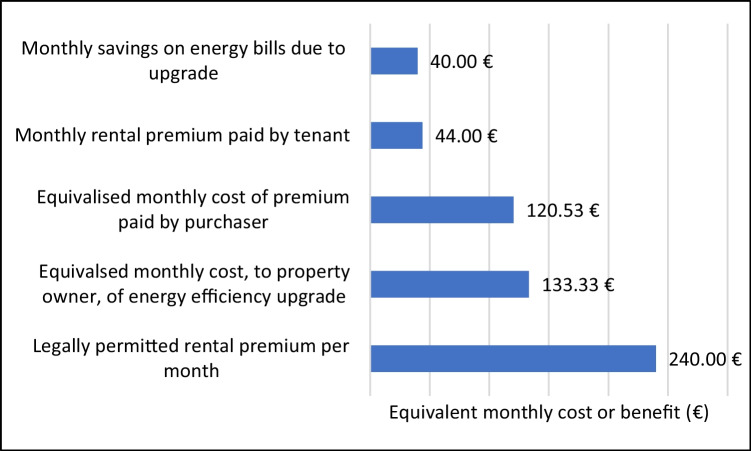
Equivalent monthly costs or benefits to property owner, purchaser and tenant, for retrofit that reduces energy intensity by 100kWh/m2/y. Equivalised monthly costs are calculated on the basis of 25-year lifetime of energy efficiency retrofit measures

Figure 5 summarises the features of the market in graphical form, showing relevant figures on an equivalised monthly basis. Starting from the bottom of the chart, the property owner is legally permitted to charge a tenant up to 240 €/month for the energy efficiency upgrade. The next bar above this reflects that fact that the upgrade costs the owner the equivalent of 133.33€/month if spread over the 25-year lifetime of the retrofit measures. If the owner sells the property (next bar up), they are unlikely to recoup their investment without subsidies, gaining an equivalent of 120.53 €/month over the 25-year lifetime. If they rent it out (next bar up), they are likely to recoup only 44.00 €/month. The tenant, who pays this premium (top bar), is likely to save only 40.00 €/month on energy consumption.

Again, it must be emphasised that these are average figures that pertain to the whole market of 1946–1979-era apartments for rent and sale in western Germany. The figures of 44.00 €/month and 120.53 €/month vary markedly depending on local markets, as shown in the next subsection, while the other three figures depend on changes over time in energy prices, costs of retrofitting, and policy on rent increases.

### Results for more regions

**Fig. 6:**
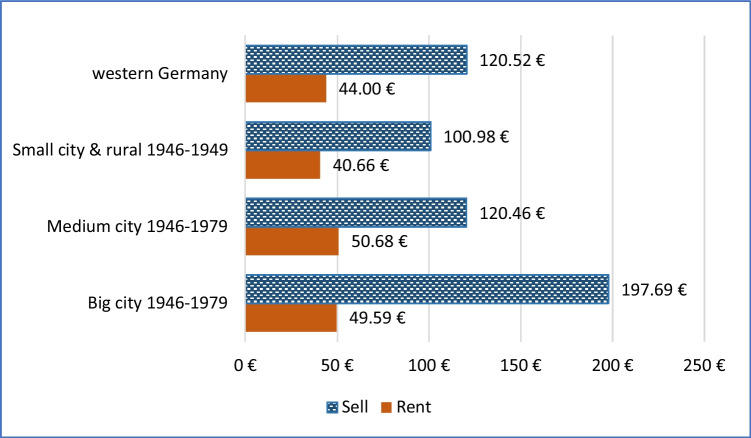
Rental premium and sales premium equivalised over 300 months, for 1946–1979-era apartments in large, medium, and small cities or rural regions, in western Germany, for a reduction of 100 kWh/m^2^/y

Figure 6 displays monthly rental premiums and sales premiums equivalised over 300 months (a 25-year lifetime of the energy-efficiency renovation measures) for 1946–1979-era apartments in western Germany as a whole, alongside those for big cities, medium cities, and small city/rural regions. In every case, the equivalised monthly sales premium is several times as large as the rental premium, indicating that, generally, a property owner gets a far higher proportion of the money they spend on energy efficiency retrofitting by selling than by renting. For big cities, this is about four times as much; for other regions, more than twice as much.

**Fig. 7:**
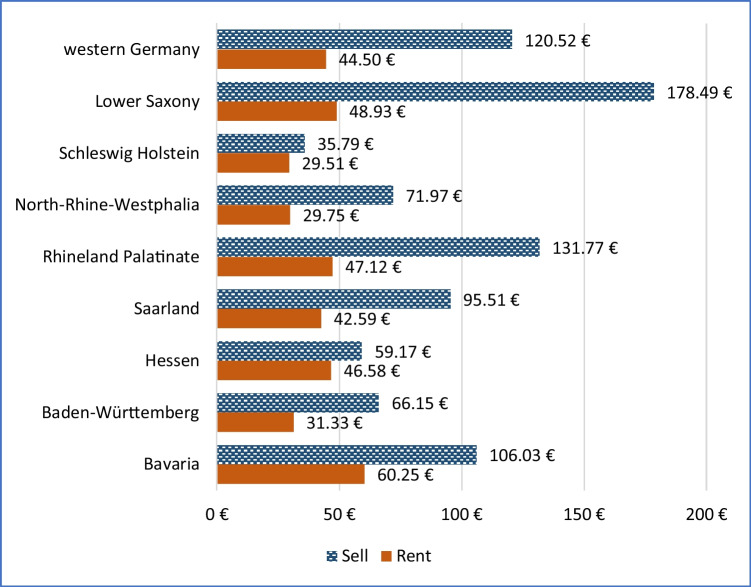
Rental premium and sales premium equivalised over 300 months, for 1946–1979-era apartments in western Germany and by state (excluding city-states Hamburg and Bremen), for a reduction of 100 kWh/m^2^/y

Figure 7 gives the same type of display for the eight federal states of western Germany that are not city states (i.e. excluding Bremen and Hamburg). Again, in every case, selling gives a far higher return on an energy efficiency investment than renting. There is much variation between states. Some of this might have to do with the spatial density of 1946–1979-era apartments compared to older buildings. Areas that were heavily bombed during the Second World War and/or expanded rapidly in population in the post-war decades will have a far higher proportion of these apartments. If these are retrofitted to high energy efficiency, their energy efficiency is closer to the average energy efficiency of the local building stock. This has not yet been investigated and would be an interesting topic for future research.

**Fig. 8:**
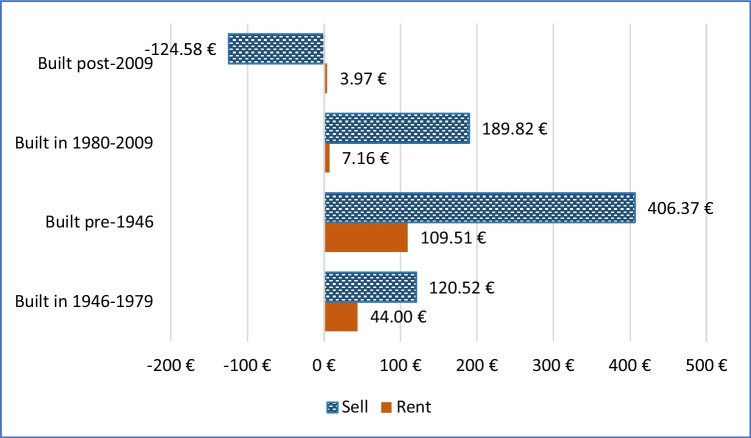
Rental premium and sales premium equivalised over 300 months, for four different cohorts of apartments in western Germany, for a reduction of 100 kWh/m^2^/y

Figure 8 gives the same type of plot for the four different cohorts of apartments.

### Did the COVID-19 pandemic make a difference?

Table 5 gives regression results, and also the means of the variables, for two extra analyses of sales data: one for the last two years before the COVID-19 pandemic 2018–2019, and one for the pandemic years 2020–2021. Linear regression result only are shown here as this makes for easy comparison of different variables’ coefficients, and the results of log-linear regressions translate to almost the same values.^6^

**Table 5:** Regression results and means of the variables for sales, for the last two years of the COVID-19 pandemic 2018–2019, and for the pandemic years 2020–2021, with percentage changes

	Regression results				Means		
Regressed against sales price	Years of sales	2018–2019 (pre-pandemic)	2020–2021 (pandemic years)	Percentage change in regression coefficients	2018–2019 (pre-pandemic)	2020–2021 (pandemic years)	Percentage change in means
**Sales price**					**242,584**	**306,783**	**26.5**
Floor area	Coefficient	2457.589	3170.154	29.0	87.1	88.4	1.5
	*t-statistic*	*57.39*	*57.98*				
**Number of bathrooms**	**Coefficient**	**6515.898**	**25,450.47**	**290.6**	1.141	1.151	0.9
	*t-statistic*	*1.64*	*5.2*				
**Energy intensity**	**Coefficient**	− **314.8894**	− **352.4539**	**11.9**	**150.2**	**148.6**	− **1.1**
	*t-statistic*	− *13.2*	− *12.42*				
Number of balconies	Coefficient	24,275.42	10,744.98	− 55.7	0.816	0.773	− 5.3
	*t-statistic*	*6.74*	*2.66*				
**Months since start**	**Coefficient**	**2794.701**	**3103.817**	**11.1**	144.2	168.3	16.7
	*t-statistic*	*13.73*	*13.02*				
Built-in kitchen	Coefficient	31,596.59	38,859.42	23.0	0.532	0.513	− 3.5
	*t-statistic*	*11.51*	*11.7*				
Basement storage	Coefficient	− 4203.626	− 7782.67	85.1	0.753	0.757	0.6
	*t-statistic*	− *1.3*	− *1.98*				
Parking available	Coefficient	7927.114	31,546.32	298.0	0.985	0.985	0.0
	*t-statistic*	*0.71*	*2.35*				
Big city	Coefficient	175,164.4	185,911	6.1	0.179	0.202	12.9
	*t-statistic*	*48.59*	*44.35*				
Medium city	Coefficient	22,167.61	6452.692	− 70.9	0.091	0.082	− 10.0
	*t-statistic*	*4.64*	*1.06*				
Constant	Coefficient	− 409,108.4	− 564,176.5				
	*t-statistic*	− *12.82*	− *12.9*				
No. of observations		11,451	9,686				
Adjusted *R*-squared		0.4132	0.4531				
*F*		0.000	0.000				

Regarding the averages (last three columns), the floor area, number of bathrooms, energy intensity, availability of parking, and basement storage of apartments in the sales market did not change significantly between these two periods. However, the average sales price increased by 26.5%, or just over 13%/y, which is consistent with, but higher than, the average long-running price increase in 2007–2021.

Regarding the regression coefficients (columns 3–5), the most interesting is the 290% increase in price premium for an extra bathroom. One could speculate that this was motivated by a sharp increase in concern for personal hygiene during the pandemic. Note, also, that the t-statistic for “number of bathrooms” is very low in 2018–2019 but rises to three times that level in 2020–2021, indicating a substantial increase in desire for an extra bathroom.

Most important for this study, the sales premium for energy intensity increased by only 11.9% between the two periods, less than half the increase in average sales price, while its t-statistic reduced slightly. Also, the 95% confidence intervals for the two periods overlap (− 361 to − 268 compared to − 408 to − 297), suggesting that the difference is not statistically significant. Hence, there might have been a small reduction in desire for energy efficiency during the pandemic, but it does not seem to have made a major difference.

Table 6 gives a similar comparison for the rental market. The mean rent increased by 6.7%, somewhat slower than the steady rental increase of about 3.7%/y in 2007–2021 (Fig. 3). The rental premium for energy efficiency increased from 0.412 to 0.457 €/month per reduced kWh/m^2^/y, or 8.4%, which was slightly higher than the average rent increase but not statistically significant (95% confidence intervals − 0.466 to − 0.358 for 2018–2019, compared to − 0.498 to − 0.396 for 2020–2021). Interestingly, in contrast to sales, the rental premium for an extra bathroom reduced during the pandemic, from pre-pandemic 120.90 €/month to 90.60 €/month, and this was statistically significant (the 95% confidence intervals did not overlap).

**Table 6:** Regression results and means of the variables for rents, for the last two years of the COVID-19 pandemic 2018–2019, and for the pandemic years 2020–2021, with percentage changes

	Regression results				Means		
Regressed against monthly basic rent	Years of sales	2018–2019 (pre-pandemic)	2020–2021 (pandemic years)	Percentage change in regression coefficients	2018–2019 (pre-pandemic)	2020–2021 (pandemic years)	Percentage change in means
Monthly basic rent					**679.4**	**725.0**	**6.7**
Supplementary rent	Coefficient	1.16	0.94	-18.9	157.3	162.0	3.0
	*t-statistic*	*32.04*	*28.28*				
Floor area	Coefficient	6.22	6.98	12.1	79.1	78.2	**-1.1**
	*t-statistic*	*79.56*	*93.54*				
**Energy intensity**	**Coefficient**	− **0.412**	− **0.447**	**8.4**	**149.3**	**146.2**	**-2.1**
	*t-statistic*	− *14.97*	− *17.13*				
Number of bathrooms	Coefficient	120.9	90.6	− 25.1	1.068	1.069	0.1
	*t-statistic*	*17*	*13.48*				
Number of balconies	Coefficient	24.6	24.9	1.0	0.764	0.744	− 2.6
	*t-statistic*	*5.93*	*6.56*				
Months since start	Coefficient	1.18	2.12	79.7	144.6	167.8	16.0
	*t-statistic*	*4.88*	*9.16*				
Built-in kitchen	Coefficient	108.7	109.6	0.8	0.410	0.458	11.7
	*t-statistic*	*30.94*	*33.95*				
Heating costs in rent	*Coefficient*	− 10.6	12.8	− 221.4	0.586	0.629	7.2
	*t-statistic*	− *2.47*	*3.21*				
Basement storage	Coefficient	22.3	10.1	− 54.8	0.754	0.753	0.0
	*t-statistic*	*5.61*	*2.71*				
Parking available	Coefficient	4.45	− 12.04	− 370.3	0.949	0.971	2.4
	*t-statistic*	*0.57*	− *1.27*				
Big city	Coefficient	229.4	240.5	4.8	0.191	0.196	2.3
	*t-statistic*	*50.57*	*57.15*				
Medium city	Coefficient	16.8	26.2	56.4	0.127	0.106	− 16.2
	*t-statistic*	*3.22*	*4.99*				
Constant	*Coefficient*	− 357.3	− 483.0	35.2			
	*t-statistic*	− *9.73*	− *11.89*				
No. of observations		18,248	18,790				
Adjusted *R*-squared		0.6262	0.6495				
*F*		0.000	0.000				

In conclusion, the pandemic did not interrupt the persistent, steep increase in sales prices nor the steady but more gradual increase in rental prices. It does not seem to have significantly increased the sales or rental premiums for energy efficiency, which are more or less consistent with the sales and rental price increases. The pandemic is associated with a very marked increase in the sales premium for an extra bathroom, but a small reduction in the rental premium for an extra bathroom. Overall, then, it seems safe to conclude that the analyses in the “Descriptive statistics”, “Regression results: premiums for energy efficiency”, “Will this market support energy-efficient renovation among 1946–1979-era apartments?”, “Results for more regions”, and “Did the COVID-19 pandemic make a difference” sections are not compromised by covering a period that precedes and includes the pandemic years.

## Conclusion and policy implications

This study has investigated the sales and rental markets in 2019–2021 for energy efficiency among apartments built in 1946–1979 in western Germany. These represent a notoriously energy-inefficient building cohort, but they are relatively homogeneous, and considerable know-how and experience have accumulated in retrofitting them to reasonably high energy efficiency standards.

The study found that, on average, the *sales* market premium for energy efficiency will only support energy efficiency retrofitting from the standpoint of the *vendor* if he or she has received standard federal subsidies for energy efficiency upgrading. However, the sales market does not support the interests of the *purchaser*, who is likely to recoup less than one-third of the purchase premium through energy savings over the lifetime of the retrofit measures. Similarly, a property owner who *retrofits and lives in* the apartment will suffer the same mismatch.

For property owners who retrofit and *rent out* an apartment, the premium is very low, about one-third the cost of retrofitting. This is especially ironic because the legally permitted rent increase after retrofitting is almost six times as high as the rental premium that the market actually supports (see Fig. 5).

For *tenants* the rental market comes close to supporting a rental premium that is largely recouped by the reductions in energy costs, but this varies from region to region.

In short, the market best supports property owners who retrofit and sell, and tenants who heat their homes to the level of full comfort recommended in the DIN standard, i.e. all rooms at least 19C all year round.

These findings vary widely between regions within western Germany. Both the sales and rental premiums are below average in small cities and rural areas, and above average in big cities (population > 400,000). By federal state, sales premiums are above average in Lower Saxony and Rhineland-Palatinate, below average in North Rhine-Westphalia, very low in Schleswig–Holstein, Hessen and Baden-Württemberg, and close to average in Saarland and Bavaria. Rental premiums are much more uniform, but somewhat below average in Schleswig–Holstein, North Rhine-Westphalia and Baden-Württemberg, above average in Bavaria and about average in the other states.

Some interesting policy implications follow from these findings. In general, the findings raise the issue of how the market could better be used as an instrument to increase the rate and depth of energy efficient renovation among this cohort of apartments. To begin with, federal government subsidies already make it economically viable, on average, for property owners to retrofit in order to sell. Second, however, in regions where sales premiums are low, a case can be made for extra subsidies from the state or municipality. Many municipalities and states already offer subsidies, but these could be more closely matched to the market conditions and finely tuned for the particular cohort of buildings. Tables 3 and 4 show that the premiums differ markedly between building cohorts, but to this author’s knowledge there are no cases of state local subsidies being adjusted specifically to suit the market needs of the building cohort. A case could also be made for more generous federal subsidies, such as longer term fixed-interest loans. There are now reforms planned to come into effect in 2025 (BMWK/BMWSB, 2022), but these are mostly aimed to support very high-efficiency renovations, which are not usually technically suited to this cohort of apartments.

Third, policymakers need to address the substantial gap between the cost of retrofitting and the rental premium (see especially Fig. 5). Civil Law §559 makes it theoretically possible for a property owner to recoup up to twice the retrofit costs through the rent, but this has failed to address the problem. The market simply does not permit such increases for new rentals (and for existing rentals it often leads to evictions and poverty—see Grossmann, 2019). Instead, there need to be incentives for property owners to “retrofit-and-rent-out”, designed to correct the market shortfall in particular regions. For example, based on values from Fig. 5, if the equivalised monthly cost of retrofitting is 1.33 €/month per reduced kWh/m^2^/y and the rental market offers only 0.44 €/month, a local subsidy amounting to the difference between these could be offered to the property owner for as long as an apartment is rented out (less any federal subsidies already received).

Fourth, as of 2022, retrofitting takes place in the context of unstable and steeply increasing interest rates, increasing construction costs and high, and unstable energy prices. The latter makes energy efficiency renovation more profitable, while the former two make its costs higher and less predictable. Policy needs to support a growing, economically efficient renovation-construction industry, while long-term fixed-interest loans for energy efficiency renovation would give property owners more confidence to renovate their buildings.

Alternatively, it could be argued that property owners have a civic duty to keep their buildings in a climate-friendly condition and must therefore bear at least a substantial portion of the loss. A number of financial models for cost sharing have been tried on a technical level (Weber & Wolff, 2018), but there needs to be deliberative policy discussion on which actors carry civic responsibility for the climate impact of residential buildings and how this responsibility should be shared.

Fifth, for tenants, policymakers need to address the small but often significant gap between the rental premium and energy cost savings, again on a region-by-region level. Whether tenants actually offset the premium through energy savings depends on their heating needs and practices as well as the size of the average gap between the premium and the cost savings (Harputlugil & de Wilde, 2021). There are calls for rental increases to be limited to actual energy savings (Weber & Wolff, 2018), but this does not address the market mismatch for new rentals: the property owner can only charge what the market offers, and the tenant has to pay what the market demands. More social housing would, of course, take pressure off the market premium.

Finally, and more generally, the study points to the need for policy to consider how the sales and rental markets for energy efficiency incentivise or dis-incentivise the market actors—property vendors, purchasers, and new tenants—in the niche area of western Germany’s post-war building boom apartments. Policymakers cannot leave it to the market, because the market is producing anomalies and injustices and, particularly in the rental market, is dis-incentivising energy efficient renovation.

## Data Availability

The data used in this work is available from the – Leibniz-Institut für irtschaftsforschung, https://www.leibniz-gemeinschaft.de/institute/leibniz-institute-alle-listen/rwi-leibniz-institut-fuer-wirtschaftsforschung.

## Footnotes

1 The author has consulted with building industry professionals and found none with experience in upgrading these properties.

2 https://www.immobilienscout24.de/?seaid=g_brand&gclid=CjwKCAjw0dKXBhBPEiwA2bmObWTDqchZz9ptBcxQQztYYkRuZLvaw2jfNd6887btYE4EdTrelwp7DBoCW6MQAvD_BwE

3 Note that the default parameter here is, effectively, rural/small city. The coefficients for big city and medium city indicate the extra sales (or rental) price due to an apartment being in a big city or medium city, respectively, compared to the price in a rural or small city setting.

4 Nevertheless, *p* values are available upon request.

5 ln (293,195) = 12.58859, add this to the regression coefficient, i.e., 0.0104, gives12.59899. e^12.59899^ = 296,260.

6 Charts of log-linear regression results are available on request.
